# Apoptin Gene Delivery by the Functionalized Polyamidoamine (PAMAM) Dendrimer Modified with Ornithine Induces Cell Death of HepG2 Cells

**DOI:** 10.3390/polym9060197

**Published:** 2017-05-29

**Authors:** Yoonhee Bae, Su Jeong Song, Ji Young Mun, Kyung Soo Ko, Jin Han, Joon Sig Choi

**Affiliations:** 1 National Research Laboratory for Mitochondrial Signaling, Department of Physiology, Department of Health Sciences and Technology, BK21 plus Project Team, College of Medicine, Cardiovascular and Metabolic Disease Center, Inje University, Busan 614-735, Korea; yoonhee-1@hanmail.net; 2 Department of Biochemistry, College of Natural Sciences, Chungnam National University, Daejeon 305-764, Korea; song.sj87@gmail.com; 3 Department of Biomedical Laboratory Science, College of Health Science, Eulji University, Seongnam, Gyeonggi-Do 461-713, Korea; mjy1026@gmail.com; 4 BK21Plus Program, Department of Senior Healthcare, Graduate School, Eulji University, Daejeon 305-764, Korea; 5 Department of Internal Medicine, Sanggye Paik Hospital, Cardiovascular and Metabolic Disease Center, Inje University, Seoul 139-707, Korea; kskomd@paik.ac.kr

**Keywords:** apoptin, nonviral gene delivery, apoptosis, polyamidoamine dendrimer, gene therapy

## Abstract

The use of tumor-specific therapeutic agents is a promising option for efficient and safe nonviral gene transfer in gene therapy. In this study, we describe the efficacy of polyamidoamine (PAMAM)-based nonviral gene delivery carriers, namely, an ornithine conjugated PAMAM (PAMAM-O) dendrimer in delivering apoptin, a tumor-specific killer gene, into human hepatocellular carcinoma (HepG2 cells) and dermal fibroblasts. We analyzed the transfection efficiency by the luciferase assay and assessed cell viability in both cell types. The transfection efficiency of the PAMAM-O dendrimer was found to be higher than that of the PAMAM dendrimer. Moreover, the cytotoxicity of the PAMAM-O dendrimer was very low. We treated both cell types with a polyplex of PAMAM-O dendrimer with apoptin, and analyzed its cellular uptake and localization by confocal microscopy. Cell cycle distribution, tetramethylrhodamine, ethyl ester (TMRE) analysis, and transmission electron microscopy imaging showed that apoptin induced cell death in HepG2 cells. We therefore demonstrated that a PAMAM-O/apoptin polyplex can be used as an effective therapeutic strategy in cancer owing to its effectiveness as a suitable nonviral gene vector for gene therapy.

## 1. Introduction

Various types of cancers plague the world today and have a major impact on society because of the associated morbidity and mortality. Cancer is characterized by uncontrolled proliferation, metastasis, cellular invasion, and angiogenesis [1,2]. The conventional therapeutic approaches for malignant tumor include surgery, chemotherapy, and radiotherapy. However, these tumor therapies have several drawbacks, such as systemic toxicity, side effects, and drug resistance, and might additionally cause tumor recurrence and metastasis [3,4]. This necessitates the development and improvement of therapeutic strategies to tackle malignant tumors. Gene therapy aims to treat human disorders by efficiently delivering genetic material such as DNA or RNA to the patient’s specific cells or tissues, without any form of biodegradation [5,6,7]. These systems can be introduced through viral or nonviral systems. Viral vectors are often used because of their very high transfection efficiency in vivo and long duration of gene expression, but their drawbacks include acute immune response, inflammation, limit of the size of the gene, and insertional mutagenesis [8,9]. To overcome the limitations of viral vectors, the field of nonviral vector systems is rapidly evolving. The commonly used nonviral vectors are liposomes, dendritic polymers, and cationic polypeptides [10,11]. Nonviral vectors as alternative gene transfer agents have many advantages, such as a lack of immunogenicity, high biocompatibility, transfer of large transgenes, and low toxicity. Among nonviral vectors, biopolymer-based gene carriers are important for therapeutic applications owing to their non-toxicity, low immunogenicity, flexible structure, and biodegradability [12,13,14]. 

Polyamidoamine (PAMAM) dendrimers are macromolecular hydrophilic cationic polymers with a highly branched structure and globular shape. PAMAM dendrimers are among the promising materials as nonviral gene delivery vectors owing to their unique characteristics such as structural flexibility for surface modification, high transfection efficiency, and low cytotoxicity [15,16]. PAMAM dendrimers have positively charged primary amine groups at the peripheral end, which form polyplexes with the negatively charged phosphate of the DNA backbone via electrostatic interactions, and these polyplexes effectively promote cellular uptake through endocytosis in various cell types [17,18]. In an earlier study, we showed that the modification of the surface functional groups of PAMAM dendrimers using basic amino acids such as histidine and arginine increased the proton buffer effect, improved the transfection efficiency, and had low cytotoxicity when used as gene delivery carriers [19]. In the present study, we grafted ornithine on the PAMAM dendrimer to develop the PAMAM-O dendrimer. Polyamines are ubiquitous and are responsible for cellular growth, proliferation, and differentiation. Many types of cancer cells have been shown in which polyamine transporters and ornithine decarboxylase are upregulated for importing exogenous polyamines and maintaining their higher cellular concentration levels [20,21]. We attempted to develop a therapeutic application using a tumor-specific killer gene and the PAMAM-O dendrimer as a nonviral gene delivery system for HepG2 cells. 

Apoptin is a 13.6 kDa basic protein rich in proline, serine, and threonine. It is derived from the chicken anemia virus, a member of the Gyrovirus genus. Importantly, apoptin selectively induces apoptosis in a variety of tumor cells without affecting non-transformed cells [22,23]. The tumor-specificity of apoptin is associated with its differential subcellular localization. In tumor cells, apoptin is predominantly localized in the nucleus, whereas in primary cells, it is mainly found in the cytoplasm. However, the precise mechanisms involved in apoptin-induced cell death remain unclear [24,25]. One mechanism might be that phosphorylation at Thr 108 of apoptin-specific kinases like cyclin-dependent kinase and PKCβ induces the apoptosis of tumor cells [26]. Second, the expression of Nur77, a nuclear orphan receptor and a member of the orphan steroid receptor family, can be relocated from the nucleus to the cytoplasm, where it regulates Nur77 binding of the anti-apoptotic protein Bcl-2, triggering the p53-independent mitochondrial death-pathway [27,28]. Apoptin is a potential selective anticancer sensor, but its applications in anticancer therapeutics pose many challenges, including the lack of efficient delivery systems [22,26]. 

We therefore aimed at developing the PAMAM-O dendrimer as an efficient gene delivery vector and determining the effectiveness of PAMAM-O/Flag-apoptin using HepG2 cells in a cancer model and dermal fibroblasts for normal cells. We hypothesized that PAMAM-O/Flag-apoptin polyplexes were released into the cytosol through endocytosis, after which the polyplexes dissociate. Apoptin gene localization and expression occur in the nuclei of cancer cells, whereas in normal dermal fibroblasts, apoptin remains in the cytosol. Phosphorylation of apoptin induced cancer-specific cell killing activity of apoptin via the intrinsic mitochondrial pathway of caspase activation.

To confirm the characteristics of the PAMAM-O dendrimer, we performed assays of polyplex formation, cell viability, and transfection efficiency. Furthermore, we examined HepG2 cells and dermal fibroblasts for anticancer activity, cell cycle distribution, tetramethylrhodamine, ethyl ester (TMRE) analysis, and transmission electron microscopy (TEM) imaging of the mitochondrial structure upon apoptin gene transfer with the PAMAM-O dendrimer. Here, we demonstrate that PAMAM-O/Flag-apoptin showed cell viability, enhanced transfection efficiency, and intracellular uptake, and could induce apoptosis via the mitochondria-mediated pathway in HepG2 cells. The results suggest that PAMAM-O/Flag-apoptin has the potential to be used in gene therapy.

## 2. Materials and Methods

### 2.1. Materials

PAMAM generation 4 dendrimer with an ethylenediamine core, *N*,* N*-diisopropylethylamine (DIPEA), *N*,*N*-dimethylformamide (DMF), PEI25KD (polyethylenimine, branched, 25KD), triisopropylsilane (TIS), and trifluoroacetic acid (TFA) were purchased from Sigma-Aldrich (Seoul, Korea). *N*-hydroxybenzotriazole (HOBt), and 2-(1H-benzotriazole-1-yl)-1,1,3,3-tetra-methyluronium (HBTU) were purchased from Anaspec (San Jose, CA, USA), and Fmoc-Orn(BOC)-OH was obtained from Novabiochem (San Diego, CA, USA). The luciferase assay kit and 5xReporter lysis buffer were purchased from Promega (Madison, WI, USA). The Micro Protein Assay kit was obtained from Pierce (Rockford, IL, USA). EZ-Cytox and EZ-LDH reagents were purchased from Daeil Lab Service (Seoul, Korea). LysoTracker Red, Nucleic acid labeling kit, Alexa Fluor 488 5-SDP ester, Trizol, Dulbecco’s modified Eagle’s medium (DMEM) supplemented with 10% fetal bovine serum (FBS), streptomycin (100 µg/mL), and penicillin (100 U/mL) were purchased from Invitrogen (Seoul, Korea). The TMRE Mitochondrial Membrane Potential Kit was purchased from Abcam (Seoul, Korea). Transcriptor Universal cDNA Master was purchased from Roche (Seoul, Korea). SYBR Green Master Mix was purchased from Bio-Rad (Seoul, Korea). 

### 2.2. Synthesis of the PAMAM-O Dendrimer 

The PAMAM-O dendrimer was synthesized as reported previously (Figure S1) [29]. In brief, amine-terminated PAMAM (generation 4) (10 mg) was dissolved in anhydrous DMF/DMSO, and the solution was reacted with 4 eq. of Fmoc-Orn(Boc)-OH, HOBt, and HBTU, and 8 eq. of DIPEA in 

DMF/DMSO (1:1, *v*/*v*) solution for 16 h at 25 °C. The conjugate was precipitated by cold ethyl ether and dissolved in 30% piperidine solution for Fmoc deprotection. The compound was precipitated in cold diethyl ether and was dissolved in a deprotection reagent (TFA:dichloromethane, 95:5, *v*/*v*) for 1 h at room temperature. The product was precipitated in cold diethyl ether and washed 3 times with an excess of diethyl ether. It was dried under nitrogen gas and further dried by a rotary evaporator. The product was characterized by ^1^H-NMR spectra (300 MHz, D_2_O). 

### 2.3. Plasmids and HepG2 Cell Culture

The luciferase reporter plasmid DNA, pJDK/Luc, pEGFP-C2, and pEGFP-C2/apoptin construct were kindly provided by Jong-Sang Park [30]. The expression vector Flag-apoptin was constructed as follows. Full-length cDNAs encoding apoptin were PCR amplified and cloned into pCMV-Flag digested with *Kpn*1 and *EcoR*1. HepG2 cells were maintained in DMEM supplemented with 10% FBS, streptomycin (100 µg/mL), and penicillin (100 U/mL) (Invitrogen).

### 2.4. Primary Culture of Dermal Fibroblasts 

Human skin samples were obtained with the written informed consent of donors, in accordance with the ethical committee approval process of the Institutional Review Board of Chungnam National University School of Medicine (CNUH 2013-04-025). Dermal fibroblasts were isolated from human foreskin tissues and were primary cultured. Skin tissues were briefly sterilized in 70% ethanol, minced, and treated with dispase overnight at 4 °C. The dermis was separated from the epidermis and placed in a culture dish for explant culture. Cells were grown in DMEM supplemented with 10% FBS (Life Technologies Corporation, Grand Island, NY, USA). Cells passaged 3–8 times were used in this study.

### 2.5. Dynamic Light Scattering and Zeta Potential Measurements

To measure the size and surface charge values, the polyplexes were prepared by mixing Flag or Flag-apoptin with PAMAM and PAMAM-O dendrimers at a weight ratio of 8. The samples were incubated for 30 min at room temperature. The solutions were diluted by adding 900 µL distilled water. The size distribution and zeta potential (surface charge) of the polyplexes were measured using an ELS-Z Analyzer (Otsuka Electronics, Osaka, Japan) and Zetasizer NanoZS (Malvern Instruments, Wocestershire, UK). 

### 2.6. PicoGreen Assay 

The Picogreen assay was performed using HEPES buffer (10 mM HEPES, 150 mM NaCI, pH 7.4) and the PicoGreen reagent (Molecular Probe) diluted in TE buffer (10 mM Tris, 1 mM EDTA, pH 7.5). The polyplex was prepared by mixing 1 µg Flag or Flag/apoptin with PAMAM and PAMAM-O dendrimers. Each sample was incubated for 30 min at room temperature. Then, the PicoGreen reagent was added to each polyplex in solution and further incubated for 2 min at room temperature. Fluorescence was measured by a filter spectrofluorometer (Quantec, Thermo Fisher Scientific Korea, Seoul, Korea). Excitation and emission wavelengths were 480 and 520 nm, respectively.

### 2.7. Agarose Gel Retardation Assay 

The agarose gel retardation assay was conducted using HEPES buffer (10 mM HEPES, 150 mM NaCl, pH 7.4). After the formation of polyplexes using PAMAM and PAMAM-O dendrimers or Flag and Flag-apoptin, the polyplexes were incubated for 30 min at 25 °C. Then, each sample was analyzed by electrophoresis on a 0.7% agarose gel containing ethidium bromide (0.5 µg/mL) at 100 V for 40 min. The gel was analyzed and photographed using a UV transilluminator (Bio-Rad, Seoul, Korea). 

### 2.8. Cytotoxicity Assay

The cytotoxicity of the polymers was measured using an EZ-Cytox assay kit (Daeil Lab Service). HepG2 cells and dermal fibroblasts were seeded in 96 well plates at a density of 1.1 × 10^4^ cells/well and incubated in 100 µL of DMEM containing 10% FBS under 37 °C and 5% CO_2_ for 24 h. The cells were treated with various concentrations of PEI25KD, PAMAM, and PAMAM-O dendrimer and then incubated for 24, 48, and 72 h. PEI25KD was used as the control group for cell viability. Thereafter, 10 µL EZ-Cytox reagent was added to each well, and the cells were incubated for an additional 2 h at 37 °C. Absorbance was measured at 450 nm using a microplate reader (VERSA max, Molecular Devices, Sunnyvale, CA, USA). The relative cell viability was calculated as a percentage relative to that of the untreated cells.

### 2.9. Lactate Dehydrogenase Release Assay

Cell membrane damage by the polymers was assessed by detecting lactate dehydrogenase (LDH) leakage into the culture medium using an LDH assay kit (Daeil Lab Service). HepG2 cells and dermal fibroblasts were seeded in a 96 well plate at a density of 1.1 × 10^4^ cells/well and incubated for 24 h. The cells were treated with various concentrations of PEI25KD, PAMAM, and PAMAM-O dendrimer and incubated for 24, 48, and 72 h. Tween 20% was used as a positive control. The supernatant was collected and centrifuged at 12,000 *g* for 3 min at room temperature. LDH release was assessed according to the manufacturer’s instructions. Absorbance was measured at 450 nm using a microplate reader (VERSA max, Molecular Devices, Sunnyvale, CA, USA). 

### 2.10. Cellular Uptake Imaging 

To measure the cellular uptake of polyplexes, HepG2 cells and dermal fibroblasts were seeded in 35 mm glass base dishes (SPL Life Science, Seoul, Korea) at a density of 5 × 10^3^ cells/well. After 24 h culture, Alexa Fluor 546-labeled Flag vector or Flag-apoptin and Alexa Fluor 488-labeled PAMAM and PAMAM-O dendrimers were prepared according to the manufacturer’s protocol. The cells were treated with the polyplexes composed of Flag or Flag-apoptin with PAMAM and PAMAM-O dendrimers at a weight ratio of 8. After further incubation for 24 h, the nuclei were stained with the NucBlue Live Cell Stain Ready probe for 5 min. The fluorescent images were analyzed using a Zeiss LSM 5 live confocal laser microscope. 

### 2.11. In Vitro Transfection Assay

For the transfection assay, HepG2 cells and dermal fibroblasts were seeded in 96 well plates at a density of 1.1 × 10^4^ cells/well and cultured for 24 h. The polyplexes were prepared by combining 1 µg of pJDK-luc with PAMAM and PAMAM-O dendrimers at various weight ratios in FBS-free media. The polyplexes were incubated for 30 min at room temperature. To compare transfection efficiency, PEI25KD was used as a positive control group (polymer/pJDK-luc weight ratio, 1) and PAMAM and PAMAM-O dendrimers were prepared with weight ratios of 1–8. After polyplex formation, cells were treated with the polyplexes and incubated for 24 h at 37 °C in complete medium containing 10% FBS. After 24 h, the medium was removed, and the cells were washed with PBS. The cells were lysed for 30 min with 50 µL of reporter lysis buffer (Promega). Luciferase activity was measured using an LB 9507 luminometer (Berthold Technology, Bad Wildbad, Germany), and protein concentrations in cell lysates were measured using the Micro BCA assay kit (Pierce). 

### 2.12. Cell Cycle Analysis

For the cell cycle phase distribution analysis, HepG2 cells and dermal fibroblasts were seeded in 6 well plates at a density of 1.3 × 10^5^/well and cultured for 24 h. The cells were transfected with the polyplex of Flag or Flag-apoptin with PAMAM and PAMAM-O dendrimers at a weight ratio of 8 and then incubated for 48 h at 37 °C. The cells were washed in 500 µL PBS, trypsinized, and centrifuged at 700 *g* for 3 min at room temperature. The cells were then fixed in 70% ice-cold ethanol at 20 °C overnight. The fixed cells were suspended twice with PBS and then treated with 5 mg/mL RNase for 30 min at room temperature. After the addition of 5 µL of propidium iodide (PI: 5 mg/mL), the samples were incubated for 10 min at room temperature. Flow cytometry analysis was performed using a FACS Calibur system (BD Biosciences, Franklin Lakers, NJ, USA) at an excitation wavelength of 488 nm and emission wavelength of 610 nm. 

### 2.13. Intracellular Trafficking Imaging

For the intracellular distribution analysis, HepG2 cells and dermal fibroblasts were seeded in 35 mm glass base dishes (SPL Life Science, Seoul, Korea) at a density of 5 × 10^3^ cells/well and incubated at 37 °C. After 24 h incubation, Alexa Fluor 488-labeled PAMAM and PAMAM-O dendrimers were prepared according to the manufacturer’s protocol. The cells were transfected with the polyplex of Flag or Flag-apoptin with Alexa Fluor 488-labeled PAMAM and PAMAM-O dendrimers at a weight ratio of 8, followed by incubation at 37 °C. After 24 h incubation, the lysosomes of the cells were stained with LysoTracker Deep Red for 30 min under 5% CO_2_ at 37 °C. The nuclei were stained with NucBlue Live Ready probes (Invitrogen) for 15 min at 37 °C. The fluorescent signal was analyzed using a Zeiss LSM 5 live confocal laser microscope.

### 2.14. Measurement of Mitochondrial Membrane Potential

Mitochondrial membrane potential (MMP) was measured using the TMRE mitochondrial membrane potential assay kit (abcam). TMRE is a lipophilic cationic red orange dye that is rapidly accumulated by active mitochondria. HepG2 cells and dermal fibroblasts were seeded in a 6 well plate at a density of 1.3 × 10^5^/well and incubated for 24 h. The cells were transfected with the polyplexes of Flag or Flag-apoptin with PAMAM and PAMAM-O dendrimers at a weight ratio of 8 and then incubated for 48 h at 37 °C. The cells were washed with PBS and incubated in complete growth medium containing 100 ng/mL TMRE for 30 min at 37 °C in the dark. We used carbonyl cyanide 3-chlorophenyl hydrazone (CCCP), a potent MMP disruptor, as the positive control. The cells were centrifuged for 3 min at 7000 *g* at room temperature, resuspended in PBS, and analyzed by flow cytometry (BD, FACS Calibur system).

### 2.15. RNA Isolation, Reverse Transcriptase PCR, and Quantitative Real-Time PCR 

For the reverse transcriptase PCR (RT-PCR) analysis, HepG2 cells and dermal fibroblasts were transfected with the polyplexes of Flag or Flag-apoptin with PAMAM and PAMAM-O dendrimers at a weight ratio of 8 and incubated for 24 h at 37 °C. Total RNA was prepared using the TRIzol reagent according to the manufacturer’s instructions (Invitrogen). RT-PCR analysis was performed using a Transcriptor Universal cDNA Master. The β-actin housekeeping gene was used as a positive control. Apoptin primers for RT-PCR and quantitative real-time PCR (q-PCR) were kindly provided by Prof. Tae-il Kim [31]. The primer sequences of β-actin were as follows: forward: 5′-ATC TGG CAC ACC TTC TAC AAT GAG CTG CG-3′, reverse: 5′-CGT CAT ACT CCT GCT TGC TGA TCC ACA TCT GC-3′. The PCR products were resolved by electrophoresis using 2% agarose gel containing ethidium bromide (0.5 µg/mL) at 100 V for 20 min. Each sample was visualized by a UV illuminator. In addition, mRNA expression was quantified using the SYBR Green Master Mix and a CF96 Real-Time PCR Detection System (Bio-Rad). The relative amount of mRNA expression was normalized to β-actin expression.

### 2.16. TEM Imaging

For ultrastructural examination, HepG2 cells and dermal fibroblasts were transfected with the polyplexes of Flag or Flag-apoptin with PAMAM and PAMAM-O dendrimers at a weight ratio of 8 and incubated for 24 h. The samples were fixed in 2.5% glutaraldehyde in 0.1 M sodium phosphate buffer (pH 7.4) at 4 °C. Each sample was then washed with the same buffer and post-fixed in 1% osmium tetroxide for 1 h. After fixation, samples were subsequently washed and then dehydrated with a graded acetone series followed by infiltration with Spurr resin. The cells were embedded into fresh Spurr resin for 24 h at 60 °C. The resulting polymerized block was trimmed and cut using an ultramicrotome (UTC; Leica, Wetzlar, Germany) with a diamond knife. The sectioned cells were mounted on copper grids and double-stained with 2% uranyl acetate and lead citrate. The grids were examined with a Hitachi H7600 Transmission Electron Microscope (Hitachi, Japan).

### 2.17. Statistical Analysis

Statistical analysis was performed using the unpaired Student’s *t*-test (*GraphPad Prism 5*). Differences between groups were considered statistically significant at *p* < 0.05 (*), *p* < 0.01 (**), and *p* < 0.00l (***).

## 3. Results and Discussion

### 3.1. Synthesis of the PAMAM-O Dendrimer

**Scheme 1:**
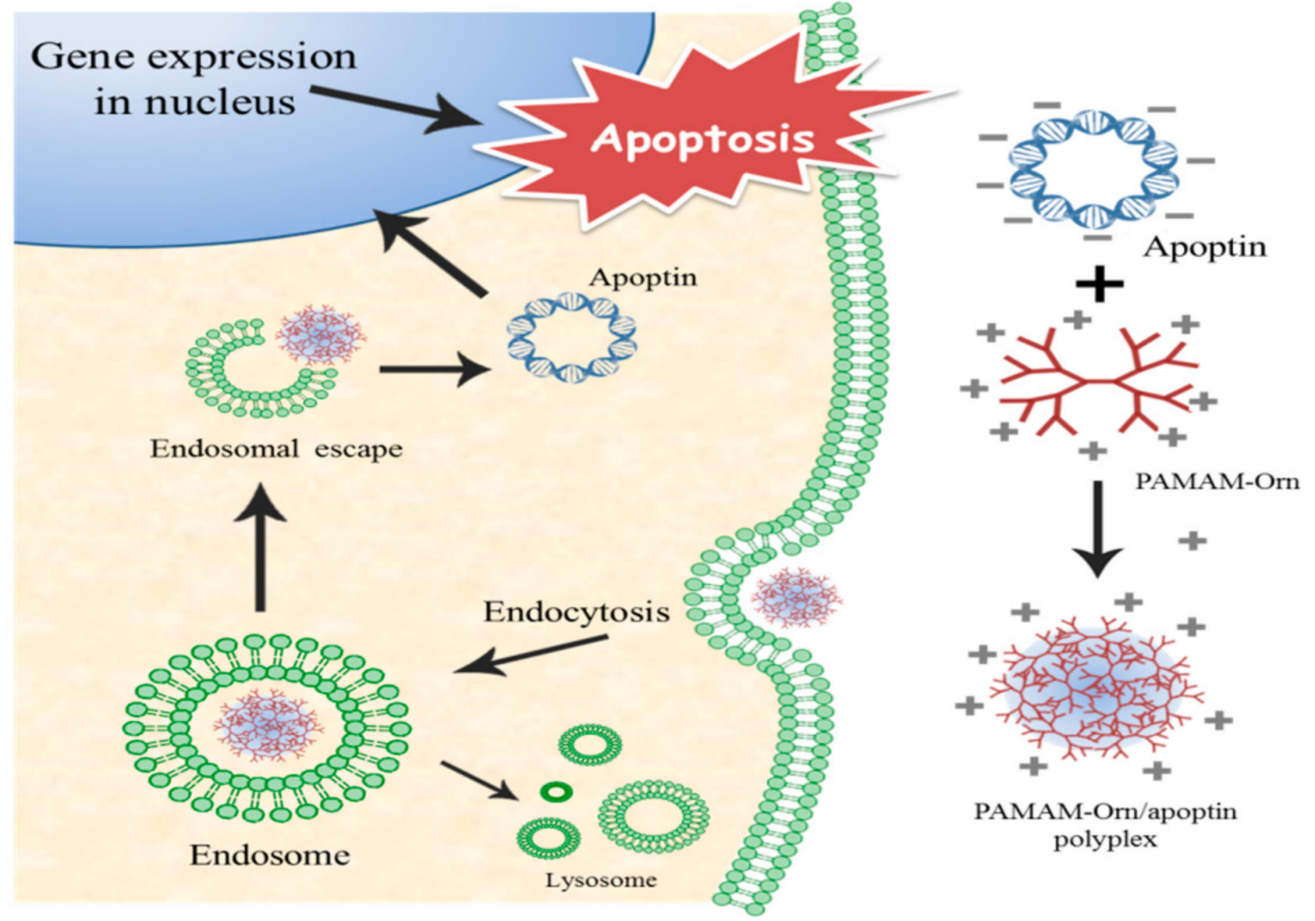
Schematic illustration of the formation of the PAMAM-O/Flag-apoptin polyplex, its release via endocytosis, targeted delivery of the apoptin gene to the nucleus, and its expression for inducing cell death in HepG2 cells.

On the basis of previous studies [20,29], we attempted to develop effective gene delivery and anti-cancer therapy systems for the human hepatocellular carcinoma cell line, HepG2. The formation of PAMAM-O/Flag-apoptin, its release through the endocytosis pathway, apoptin gene accumulation in the nucleus, and apoptin-induced cell death are schematically presented in Scheme 1. The synthesis of the amine-terminated PAMAM-O dendrimer was characterized by ^1^H-nuclear magnetic resonance (NMR) spectroscopy (Figure S2). The ^1^H NMR data suggest that the final yields of the PAMAM-O dendrimer were usually over 95% of the surface primary amines of the PAMAM dendrimer.

### 3.2. Polyplex Formation by Polymers

As a cationic polymer, the PAMAM dendrimer must form polyplexes with plasmid DNA (pDNA) via electrostatic binding between the positively charged amine of the dendrimer and the anionic phosphate backbone of DNA, which is a very important first step for efficient gene delivery [18].

**Figure 1:**
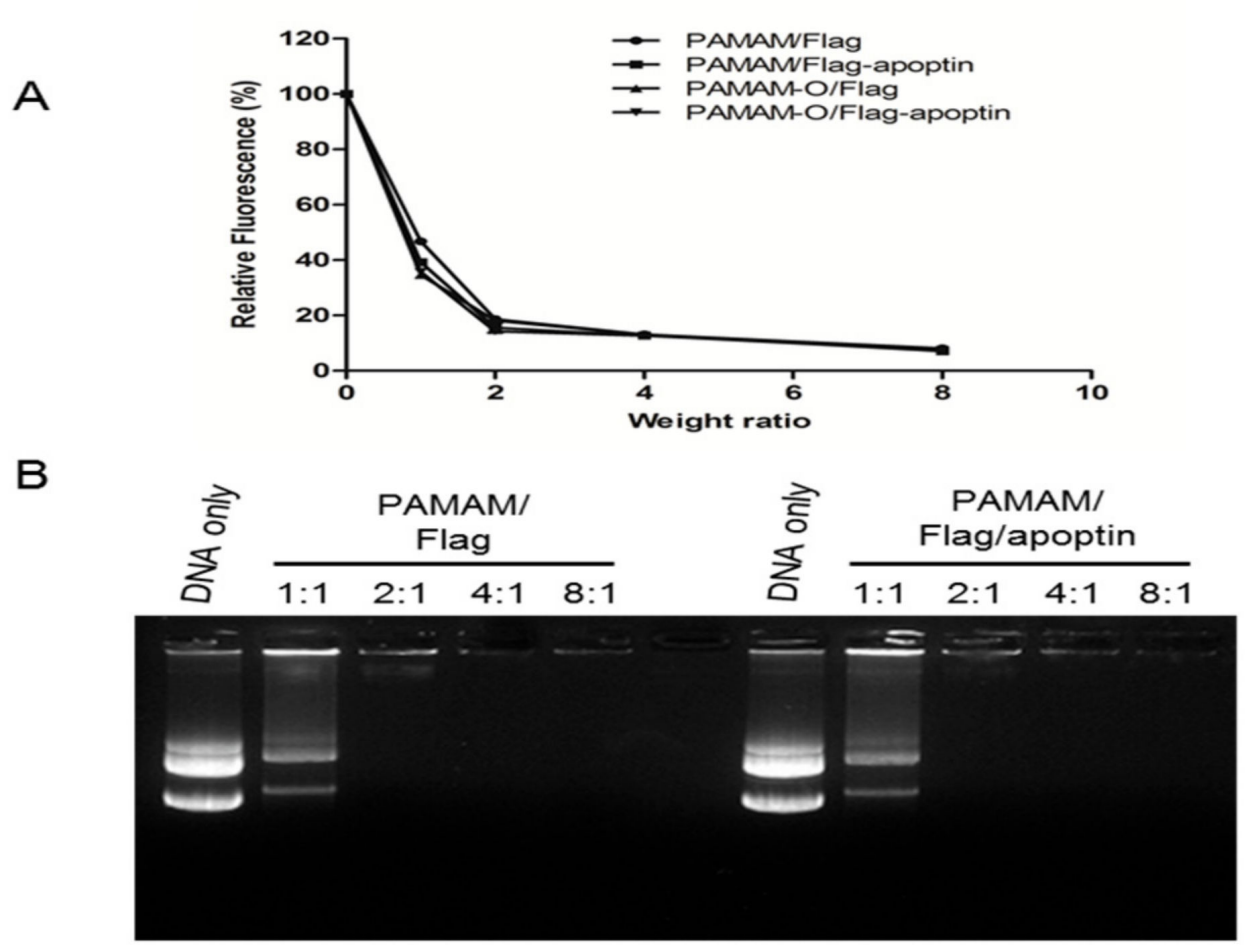

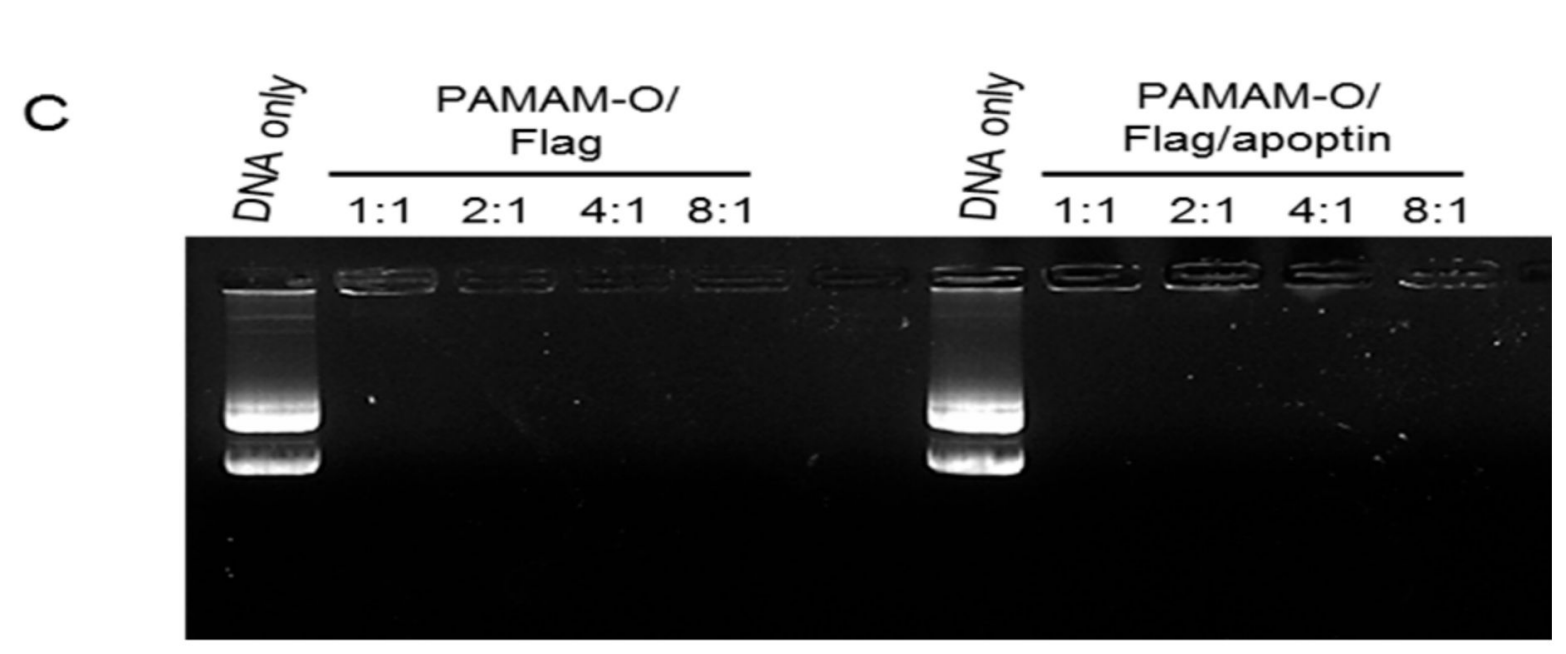
PicoGreen assay (**A**) and agarose gel retardation (**B**,**C**) of Flag or Flag-apoptin binding by the PAMAM-O dendrimer. Polyplexes were prepared with PAMAM and PAMAM-O dendrimers with Flag or Flag-apoptin at various weight ratios (0, 1, 2, 4, and 8). The signal intensity was normalized to 100% at a weight ratio of 0 (Flag only). Data represent mean ± standard deviation (*n* = 3).

**Figure 2:**
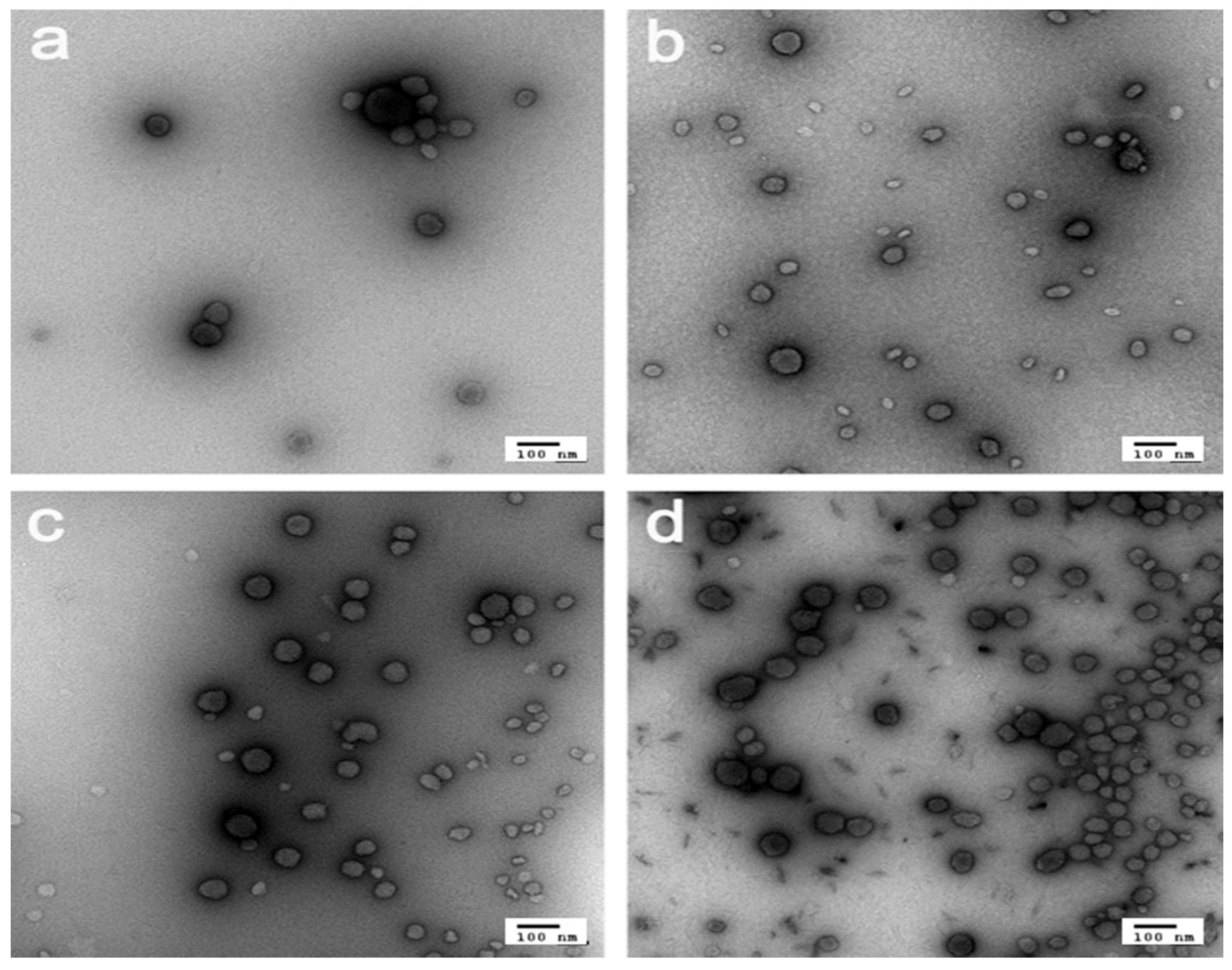
TEM images of polymer/Flag or Flag-apoptin polyplexes. (**a**) PAMAM/Flag polyplexes; (**b**) PAMAM/Flag-apoptin polyplexes; (**c**) PAMAM-O/Flag polyplexes; and (**d**) PAMAM-O/Flag-apoptin polyplexes. The scale bar is 100 nm.

To confirm previous findings regarding polyplex formation of the PAMAM-O dendrimer, the PicoGreen assay was performed. The polyplexes of PAMAM and PAMAM-O dendrimers were incubated with Flag or Flag-apoptin at weight ratios of 1 to 8. As shown in Figure 1, the pDNA polyplexes were condensed at weight ratios of 1 for PAMAM and PAMAM-O dendrimers with Flag or Flag-apoptin. In addition, an agarose gel retardation assay was performed under the same conditions as those of the PicoGreen assay. As shown in Figure 1B,C, compact DNA polyplexes showed a weight ratio of 1 for each polymer with Flag or Flag-apoptin. Thus, PAMAM-O dendrimers could effectively form polyplexes with pDNA. Also, to physically characterize the hydrodynamic and zeta potential values of each polymer, the polyplexes composed of the PAMAM and PAMAM-O dendrimers with Flag or Flag-apoptin were analyzed (Table 1). In addition, morphologies of the polyplexes of PAMAM and PAMAM-O dendrimers with Flag or Flag-apoptin at a weight ratio of 8 were examined by TEM imaging. As shown in Figure 2, each polyplex exhibited spherical morphology. These results suggest that the PAMAM-O dendrimer has potential as a gene delivery vector in vitro.

### 3.3. Cytotoxicity Assay

**Figure 3:**
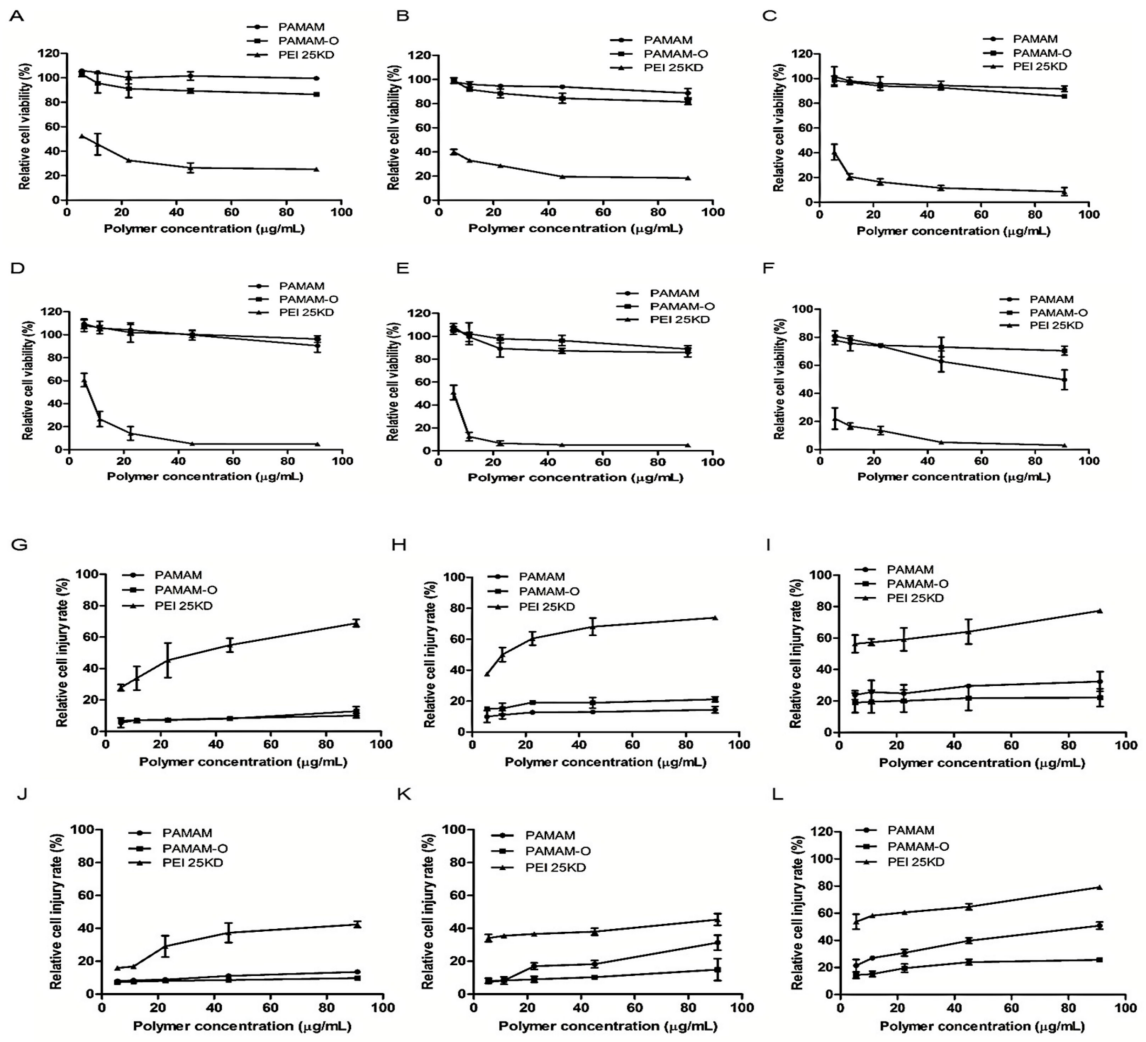
Cell viability assay for the PAMAM and PAMAM-O dendrimers. HepG2 cells were treated with different doses of PAMAM and PAMAM-O dendrimers. After 24 h (**A**); 48 h (**B**); and 72 h (**C**), cell viability was measured by the EZ-Cyto Reagent. Dermal fibroblasts were treated as in panels A, B, and C. After 24 h (**D**); 48 h (**E**); and 72 h (**F**), cell viability was measured by the WST assay. Data represent the mean ± standard deviation (*n* = 3) of three independent experiments. HepG2 cells were treated as in panels A, B, and C. At 24 h (**G**); 48 h (**H**); and 72 h (**I**) after incubation, cell viability was analyzed using lactate dehydrogenase (LDH) assays. Dermal fibroblasts were treated as in panels D, E, and F. At 24 h (**J**); 48 h (**K**); and 72 h (**L**) after incubation, cell viability was analyzed using LDH assays. Data represent the mean ± standard deviation (*n* = 3) of three independent experiments.

Cationic polymers such as PEI have shown cytotoxic effects when used as gene delivery vectors because these polymers can damage the phospholipid cell membrane. This effect is associated with surface charge density, polymer molecular weight, cell type, and structural features of each modified polymer [32,33]. The cytotoxicity of the PAMAM and PAMAM-O dendrimers in HepG2 cells and dermal fibroblasts was measured using the EZ-Cytox cell viability assay kit. PEI25KD was used as the positive control. PEI25KD showed dramatic cytotoxicity. HepG2 cells and dermal fibroblasts were treated with each delivery vector for 24 and 48 h, depending on the concentration. As shown in Figure 3A–F, the cytotoxicity of the PAMAM-O dendrimer was much lower than that of PEI25KD, even as the cationic polymer concentration increased. Moreover, dermal fibroblasts were incubated with polymers for 72 h. The PAMAM dendrimer was more toxic than the PAMAM-O dendrimer when the polymer concentration was increased. The PAMAM-O dendrimer displayed high cell viability in both cell types as expected, and it was also found to exhibit biocompatibility in both cell types. To further confirm this, the LDH assay was performed. HepG2 cells and dermal fibroblasts were treated with PEI25KD and PAMAM and PAMAM-O dendrimers, and were then cultured in concentration-and time-dependent manners. After 24, 48, and 72 h incubation, the PAMAM-O dendrimer showed a much lower cell injury rate than that of PEI25KD when the polymer concentration and time were increased (Figure 3G–L). We assume that the introduction of ornithine residues at the peripheral primary amines of PAMAM can provide more biocompatible moieties of the polymers by decreasing the cationic charge density, which should cause less destructive interaction with cells in comparison with PEI25KD. These results suggest that due to enhanced transfection efficiency, the PAMAM-O dendrimer could be a promising vector for gene delivery. 

### 3.4. Transfection Efficiency In Vitro

**Figure 4:**
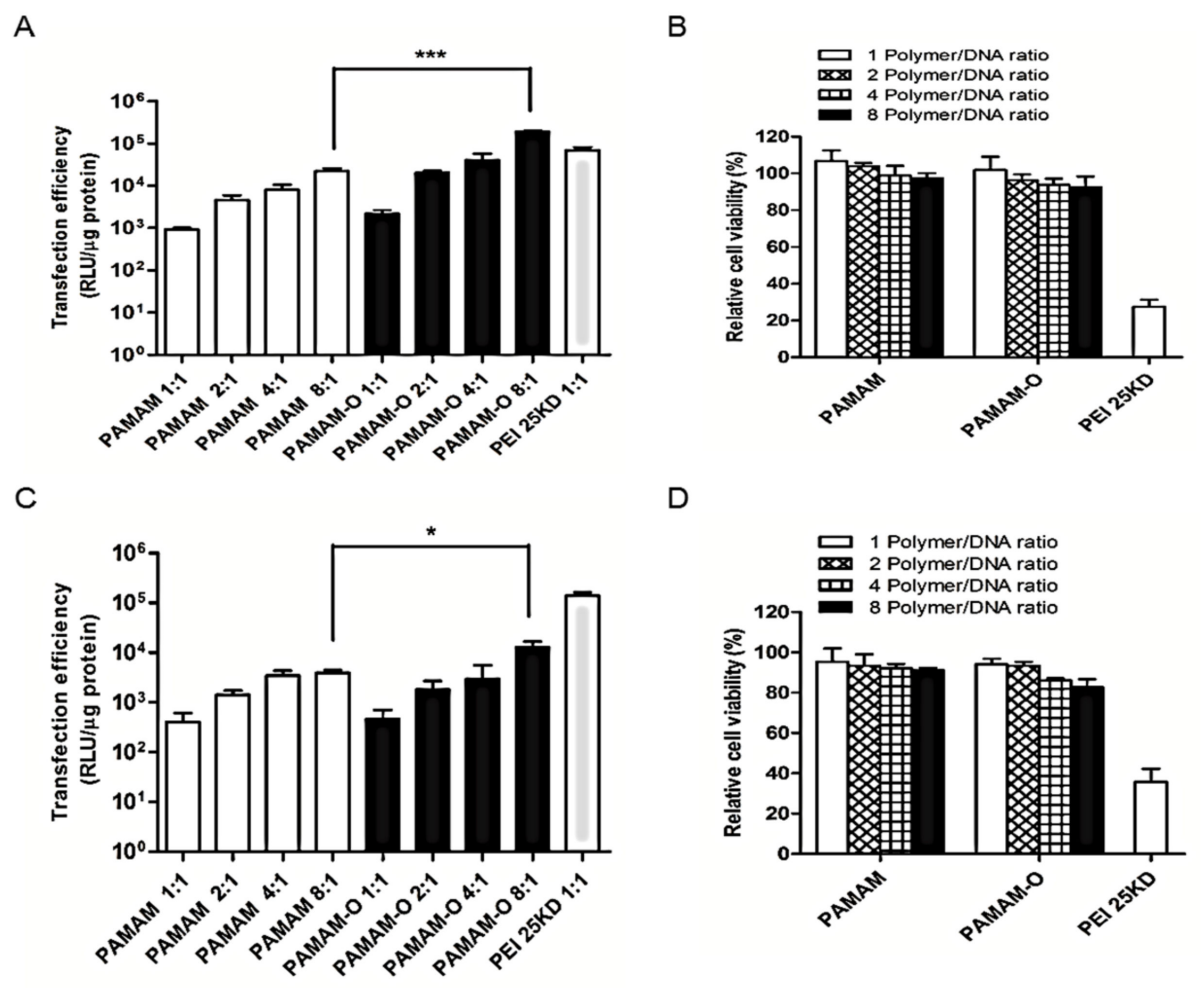
Transfection efficiency of the PAMAM-O dendrimer. (**A**) HepG2 cells were transfected with pJDK/Luc polyplexed with PAMAM and PAMAM-O dendrimers at different weight ratios (1, 2, 4, and 8). Luciferase activity was measured using Lumat LB 9507, and PEI25KD was used as a positive control. Data represent the mean ± standard deviation (*n *= 3) of three independent experiments. Asterisks show statistical significance. Unpaired *t*-test: *** *p *< 0.001; (**B**) Cells were treated at the same conditions as in panel A, and cell viability was measured by the WST assay. Data represent the mean ± standard deviation (*n* = 3) of three independent experiments; (**C**) Dermal fibroblasts were transfected at the same conditions as in panel A. Data represent mean ± standard deviation (*n* = 3) of three independent experiments. Asterisks show statistical significance. Unpaired *t*-test: * *p *< 0.05; (**D**) Cells were treated at the same conditions as in panel C, and cell viability was measured by the WST assay. Data represent the mean ± standard deviation (*n *= 3) of three independent experiments.

A previously reported PAMAM-O dendrimer has shown high efficiency as a gene delivery carrier [20]. Further experiments were performed in HepG2 cells and dermal fibroblasts. The transfection efficiency of PAMAM and PAMAM-O dendrimers was measured in both cell types using luciferase activity. To perform transfection experiments, cells were incubated with each polyplex at weight ratios of 1 to 8. At 24 h post-transfection, polyplex-treated cells were assayed for reporter gene expression in relative light units (RLU) per microgram of protein. As shown in Figure 4A, in HepG2 cells, the transfection efficiency level of the PAMAM-O dendrimer was significantly higher than that of the PAMAM dendrimer at each weight ratio tested. In dermal fibroblasts, the transfection efficiency level of the PAMAM-O dendrimer was not superior to that of the PAMAM dendrimer.

Furthermore, cell viability assays were examined under the same conditions as the transfection experiments. As shown in Figure 4B,D, for HepG2 cells, regardless of the polymer concentrations, the PAMAM-O dendrimer showed a higher cell viability than that of the PEI control. In addition, in dermal fibroblasts, treatment with PAMAM and PAMAM-O dendrimers resulted in negligible cytotoxicity. These results suggest that the PAMAM-O dendrimer can be used for further experiments, and that it is an effective vector for gene delivery in tumor cell culture systems. 

### 3.5. Expression of PAMAM-O/Flag-Apoptin Polyplexes In Vitro

**Figure 5:**
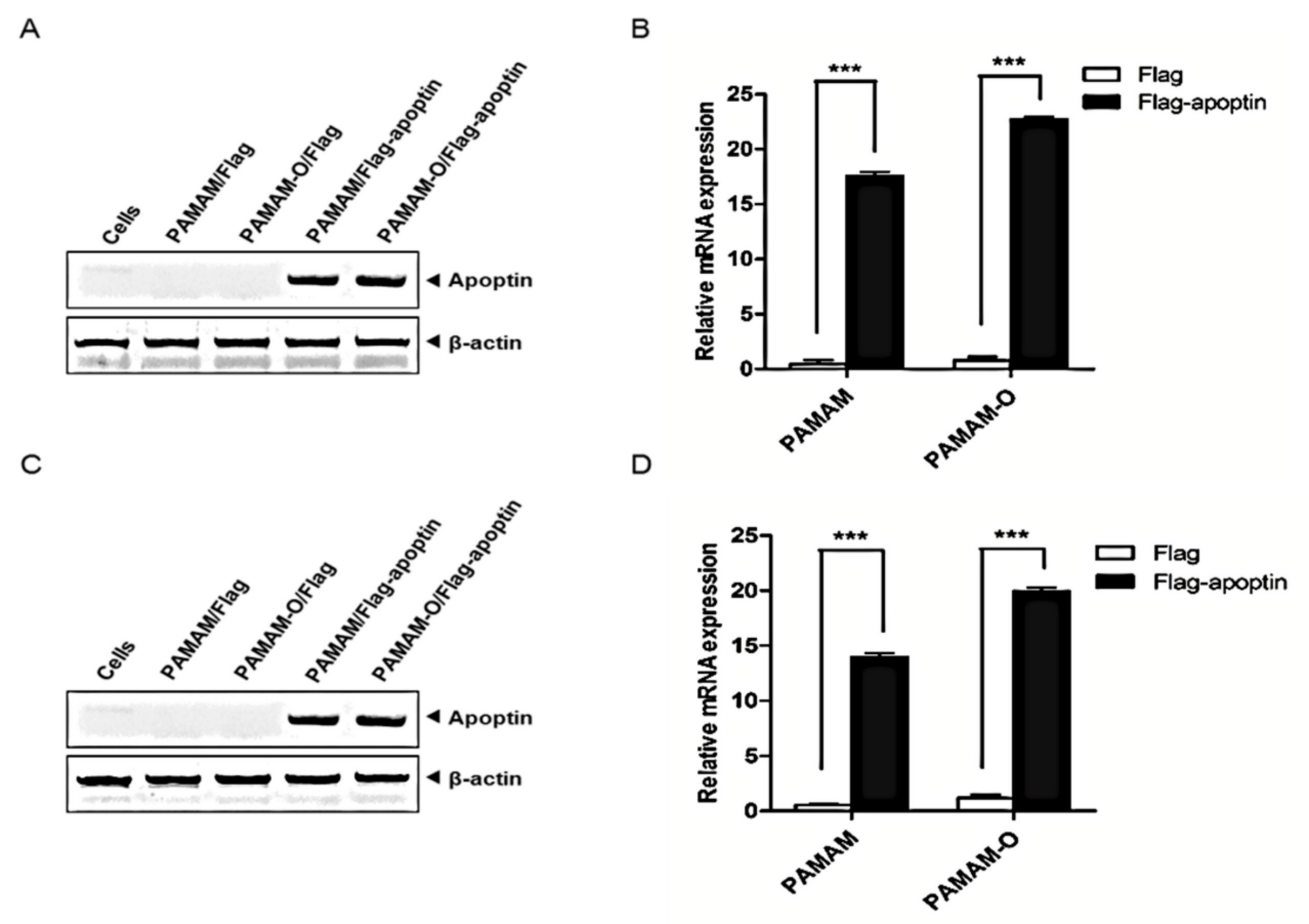
Apoptin gene expression of the PAMAM-O dendrimer. HepG2 cells (**A**) and dermal fibroblasts (**C**) were transfected with polyplexes of PAMAM and PAMAM-O dendrimers and 1 µg of Flag or Flag-apoptin (polymer:DNA = 8:1). At 24 h post transfection, apoptin gene expression was analyzed using RT-PCR (**A**,**C**) and q-PCR analysis (**B**,**D**). Data represent mean ± standard deviation (*n* = 3). Asterisks show statistical significance. Unpaired *t*-test: *** *p *< 0.001.

To examine apoptin gene expression, HepG2 cells and dermal fibroblasts were transfected with PAMAM/Flag or PAMAM/Flag-apoptin and PAMAM-O/Flag or PAMAM-O/Flag with 1 µg of apoptin at a weight ratio of 8. At 24 h post-transfection, total RNA was isolated and analyzed by RT-PCR and q-PCR experiments. The house keeping gene β-actin is widely used for normalizing gene expression at both mRNA and protein levels [34], therefore, it was used as a positive control (Figure 5A–D).The gene was expressed in all polyplexes without polymer treatment. Interestingly, apoptin gene expression significantly increased in cells treated with polyplexes of apoptin with PAMAM and PAMAM-O dendrimers. 

### 3.6. Intracellular Distribution of PAMAM-O/Flag or Flag-Apoptin Polyplexes

**Figure 6:**
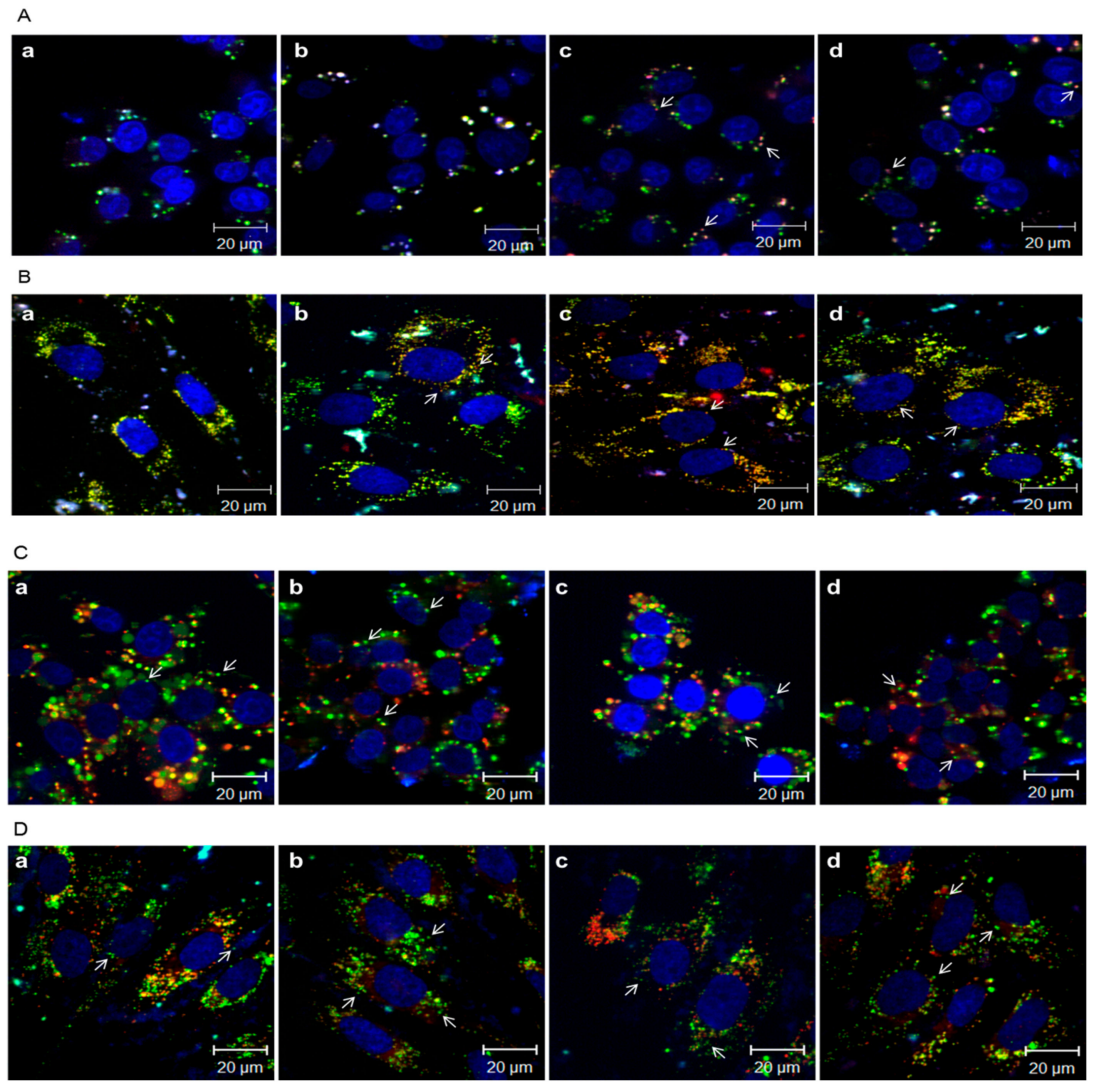
Cellular localization of the PAMAM-O dendrimer. HepG2 cells (**A**) and dermal fibroblasts (**B**) were transfected with Alexa Fluor 532-labeled 1 µg of Flag or Flag-apoptin and Alexa Fluor 488-labeled PAMAM and PAMAM-O dendrimers at a weight ratio of 8. The micrographs indicate (**a**) PAMAM/Flag; (**b**) PAMAM/Flag-apoptin; (**c**) PAMAM-O/Flag; and (**d**) PAMAM-O/Flag-apoptin, 24 h after transfection. Flag or Flag-apoptin was stained by Alexa Fluor 532 and then visualized by confocal microscopy. The nuclei and polymers were stained with NucBlue Live Ready Probes and Alexa 488 (green). Arrows indicate Flag or Flag-apoptin labeled with Alexa Fluor 532 dye. Scale bar: 20 µm. HepG2 cells (**C**) and dermal fibroblasts (**D**) were treated at the same conditions as in panels A and B. The micrographs indicate (**a**) PAMAM/Flag; (**b**) PAMAM/Flag-apoptin; (**c**) PAMAM-O/Flag; and (**d**) PAMAM-O/Flag-apoptin. Lysosomes were stained with LysoTracker Deep Red (red). The nuclei and polymers were stained with NucBlue Live Ready Probes and Alexa 488 (green). Arrows indicate PAMAM and PAMAM-O labeled with Alexa Fluor 488 dye. Scale bar: 20 µm.

The first step to consider in gene expression is intracellular uptake, in which polyplexes enter the plasma membrane via the cell surface by electrostatic binding between cationic polymers and negatively charged plasmid DNA [18]. To investigate the ability of the PAMAM-O dendrimer to efficiently uptake 1 µg of pDNA, Alexa Fluor 488-labeled PAMAM and PAMAM-O dendrimers were polyplexed with Alex Fluor 546-labeled 1 µg of Flag or Flag-apoptin at a weight ratio of 8 and were transfected into HepG2 cells and dermal fibroblasts. The intracellular distribution of the polyplexes was determined by confocal microscopy after 24 h incubation with each polyplex at 37 °C. The polyplexes were found to escape from the endosome into the cytosol, followed by accumulation in the cytoplasm or near the peri-nuclear region (Figure 6A,B). Red fluorescent spots inside the nucleus were not observed for any of the polymers because of partial degradation of pDNA and difficulty in escaping endolysosomal degradation [35]. 

To confirm the intracellular distribution of the PAMAM-O dendrimer, intracellular distribution was analyzed using LysoTracker Red, a pH-sensitive fluorescent dye used for tracking intracellular acid compartments in living cells. HepG2 cells and dermal fibroblasts were incubated with Alexa-labeled PAMAM and PAMAM-O dendrimers with 1 µg of Flag or Flag-apoptin at a weight ratio of 8. After 24 h transfection, the cells were treated with LysoTracker Red for 30 min at 37 °C. As shown in Figure 6C, D, each polyplex showed co-localization of LysoTracker (red) and the PAMAM-O dendrimer (green) in the cells, indicating that although the PAMAM-O dendrimer did not demonstrate very high endosomal escape efficiency in confocal imaging, the tertiary amines of the PAMAM-O dendrimer have proton buffering capacity, which is considered to contribute to endosomal escape of polyplexes in the cytosol for efficient transfection. Therefore, the formation of polyplexes with Flag or Flag-apoptin promoted their escape from endosomes via endocytosis, after which the polyplexes dissociated, allowing the apoptin gene to enter the nucleus for its expression. These findings imply that PAMAM-O dendrimer-mediated transfection was highly efficient and therefore has a potential therapeutic application as a gene delivery vector. 

### 3.7. Antitumor Activity of the PAMAM-O/Flag-Apoptin Polyplex In Vitro 

**Figure 7:**
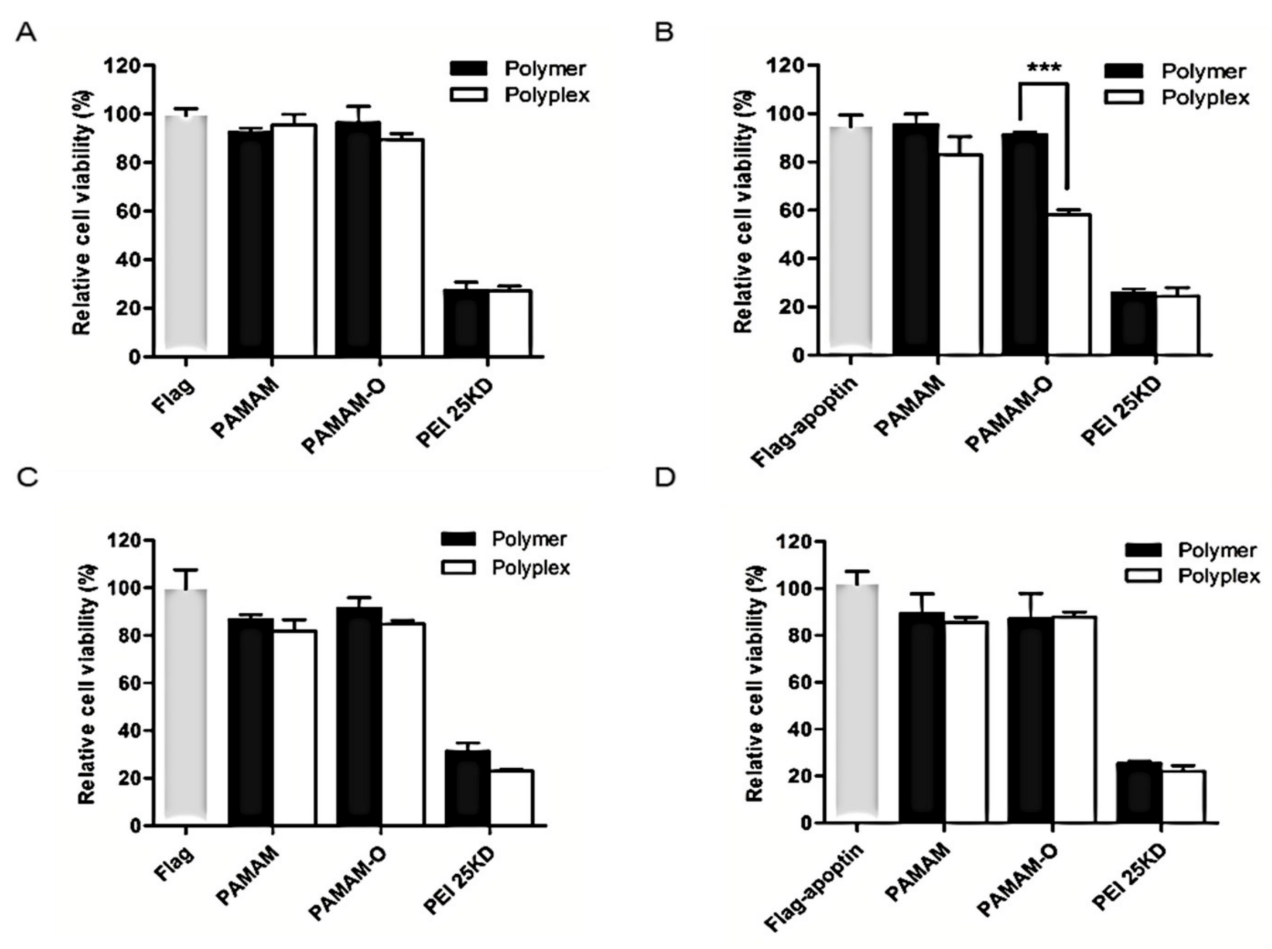
Apoptin-induced apoptosis by the PAMAM-O dendrimer. (**A**,**B**) Effect on cell viability by PAMAM-O/Flag or 1 µg of Flag-apoptin. HepG2 cells were transfected with polyplexes of PAMAM and PAMAM-O dendrimers and Flag or Flag-apoptin. After 48 h, cell viability was analyzed using the WST assay. Data represent the mean ± standard deviation (*n* = 3). Asterisks show statistical significance. Unpaired *t*-test: *** *p *< 0.001; (**C**,**D**) Dermal fibroblasts were transfected at the same conditions as in panels A and B. After 48 h, cell viability was analyzed using the WST assay. Data represent mean ± standard deviation (*n* = 3).

Apoptin induces cell death by apoptosis in tumor cells, but not in normal cells [25,36]. To investigate the cell viability of Flag or Flag-apoptin polyplexed with the PAMAM-O dendrimer using the EZ-Cytox cell viability assay kit, HepG2 cells and dermal fibroblasts were transfected with PAMAM and PAMAM-O dendrimers polyplexed with Flag or Flag-apoptin for 48 h. As shown in Figure 7A,B, both PAMAM/Flag and PAMAM-O/Flag-treated cells were viable. Interestingly, PAMAM-O/Flag-apoptin treatment showed lower cell viability than that by PAMAM-O/Flag treatment in HeG2 cells. In contrast, a normal cell line of dermal fibroblasts was viable after treatment with the PAMAM-O dendrimer and 1 µg of Flag or Flag-apoptin polyplexes (Figure 7C, D). 

**Figure 8:**
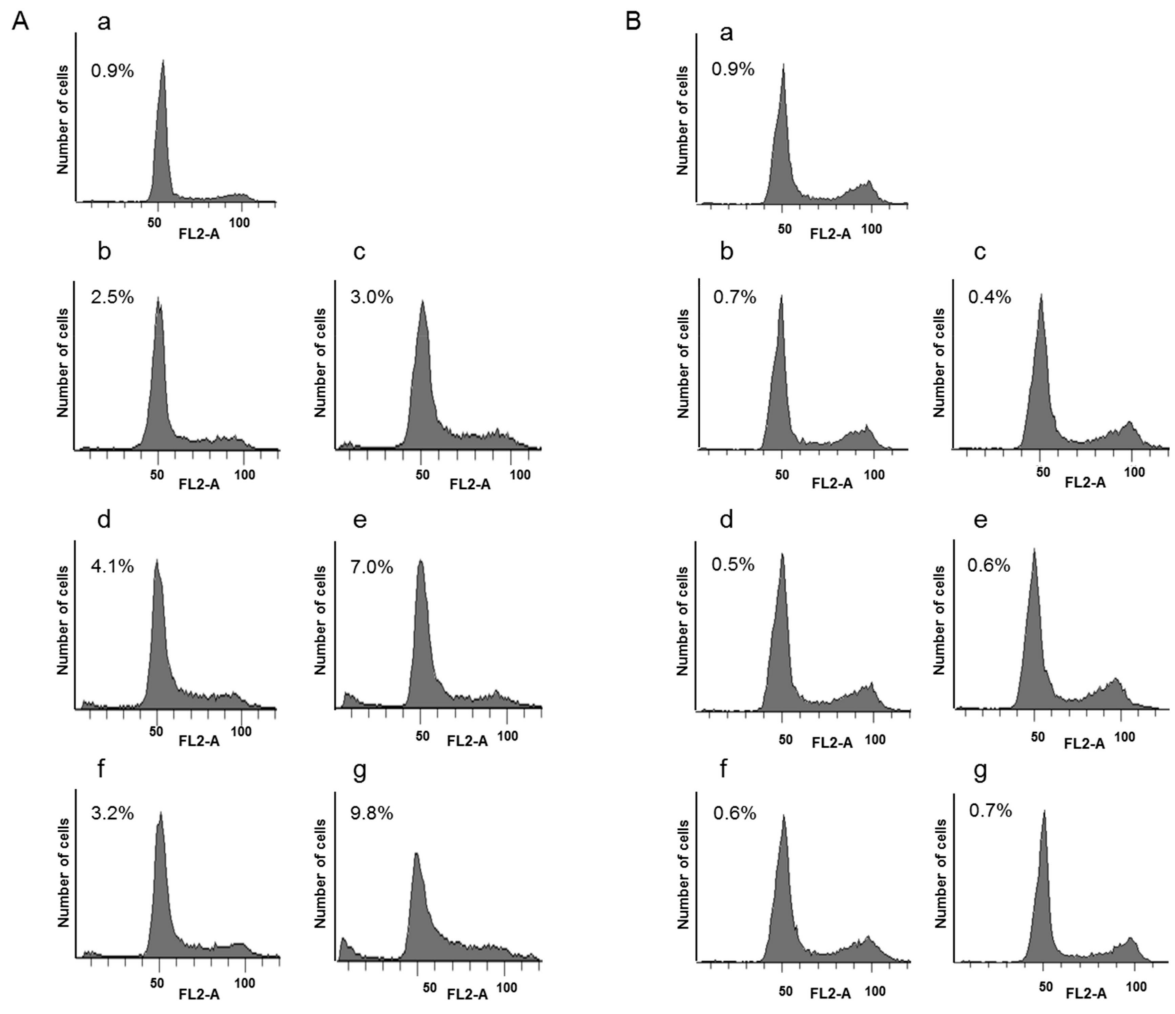
Antitumor activity of the PAMAM-O/Flag-apoptin polyplex in vitro. HepG2 cells (**A**) and dermal fibroblasts (**B**) were transfected with polyplexes with PAMAM and PAMAM-O dendrimers and 1 µg of Flag or Flag-apoptin. After 48 h incubation, the cells were harvested, and the cell cycle distribution was assessed using flow cytometry after propidium iodide (PI)-staining of nuclei. The micrographs indicate (**a**) control without polymer or polyplex; (**b**) Flag; (**c**), Flag-apoptin; (**d**) PAMAM/Flag; (**e**) PAMAM/Flag-apoptin; (**f**) PAMAM-O/Flag; and (**g**) PAMAM-O/Flag-apoptin. A total of 10,000 cells were counted for DNA content measurement. Apoptosis was measured as the percentage of the total cell population with Sub-G_0_/G_1_ DNA content (upper left corners of each graph).

Next, to investigate whether PAMAM-O/Flag-apoptin affected the cell cycle distribution, Fluorescence-activated cell sorting (FACS) analysis was performed after propidium iodide (PI) staining. HepG2 cells and dermal fibroblasts were transfected with PAMAM/Flag or PAMAM/Flag-apoptin and PAMAM-O/Flag or PAMAM-O/Flag-apoptin. After transfection for 48 h (Figure 8A,B), PAMAM-O/Flag-apoptin (9.8%) was higher than PAMAM-O/Flag (3.2%) in the sub-G1 cell population. Interestingly, PAMAM-O/Flag-apoptin treatment resulted in apoptin-induced cell death in HepG2 cells. However, according to the cell cycle distribution, PAMAM-O/Flag-apoptin treatment did not result in apoptosis in dermal fibroblasts. 

### 3.8. Effect on the Mitochondrial Membrane Potential during Apoptin-Induced Cell Death by the PAMAM-O/Flag-Apoptin Polyplex In Vitro

**Figure 9:**
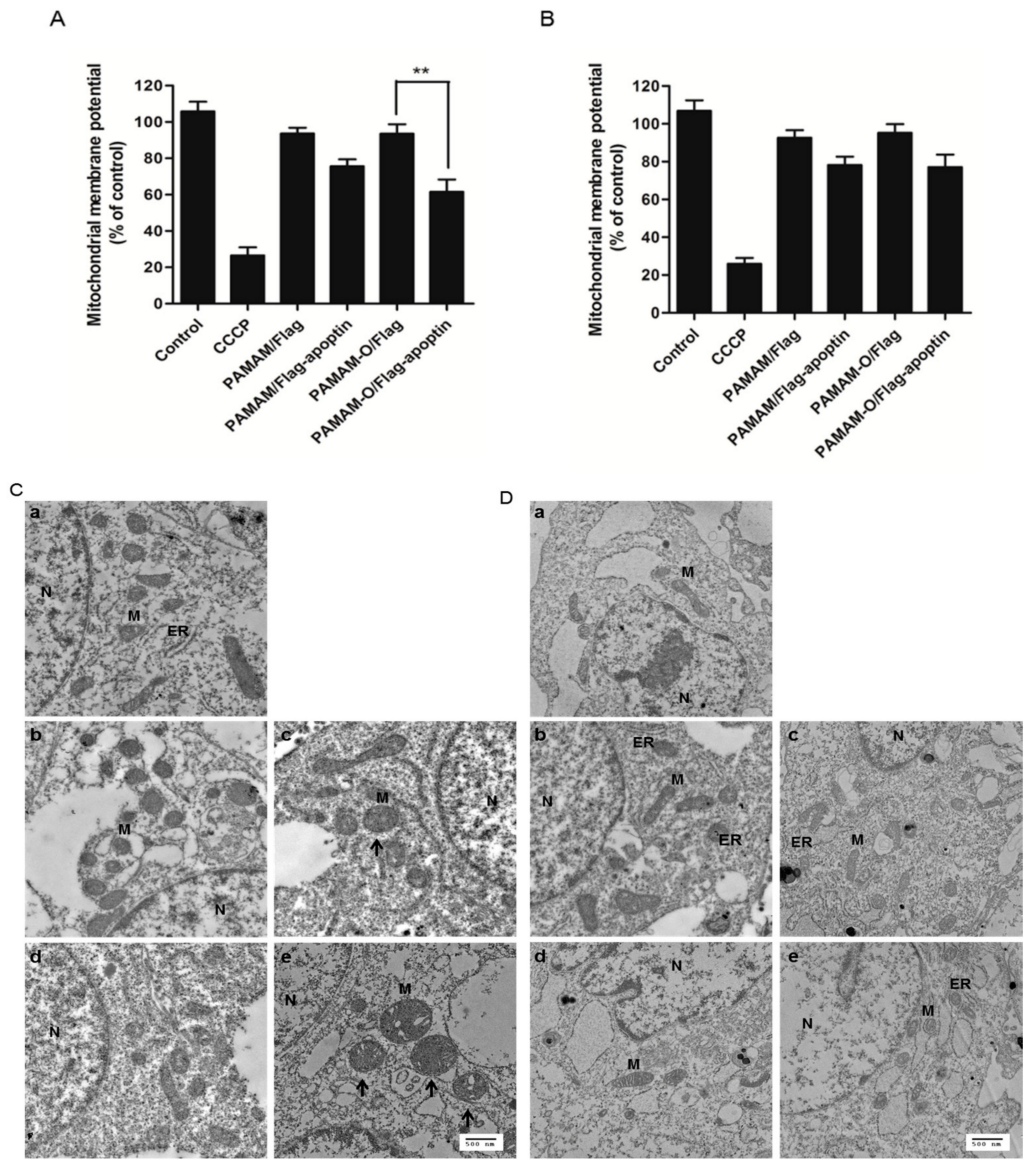
Mitochondria-mediated apoptosis by the PAMAM-O/Flag-apoptin polyplex. HepG2 cells (**A**) and dermal fibroblasts (**B**) were transfected with polyplexes of PAMAM and PAMAM-O dendrimers and 1 µg of Flag or Flag-apoptin. The cells were stained with Tetramethylrhodamine, ethyl ester (TMRE), and the mitochondrial membrane potential (MMP) was analyzed by Fluorescence-activated cell sorting (FACS). The number in the bottom right quadrant represents the percentage of MMP loss. Carbonyl cyanide 3-chlorophenylhydrazone (CCCP) was used as the positive control. Asterisks show statistical significance. Unpaired *t*-test: ** *p *< 0.01. HepG2 cells (**C**) and dermal fibroblasts (**D**) were treated as in panels A and B. TEM imaging of mitochondrial structural changes: (**a**) control cell without polyplexes; (**b**) PAMAM/Flag; (**c**) PAMAM/Flag-apoptin; (**d**) PAMAM-O/Flag; and (**e**) PAMAM-O/Flag-apoptin. N: nuclei, M: mitochondria, and ER: endoplasmic reticulum. Black arrows indicate changes in mitochondrial morphology. The scale bar is 500 nm.

Next, we asked whether apoptin-induced cell death triggers the mitochondria-mediated apoptotic pathway. The mitochondria-dependent apoptotic pathway is activated by cell death stimuli such as UV, gamma radiation, and anti-cancer drugs, leading to a loss of mitochondrial membrane potential (MMP), inhibition of ATP production, and generation of reactive oxygen species (ROS) by cellular damage [37]. This indicates that the cell death effect by PAMAM-O/apoptin is caused by a decline in MMP. Tetramethylrhodamine, ethyl ester (TMRE) is a positively charged, mitochondrial specific dye, and the red fluorescence intensity is associated with the strength of MMP [38,39]. We therefore used TMRE staining to measure the MMP in HepG2 cells and dermal fibroblasts transfected with PAMAM/Flag or PAMAM/Flag-apoptin and PAMAM-O/Flag or PAMAM-O/Flag-apoptin and then incubated for 48 h. We examined the change in MMP using FACS analysis based on the TMRE assay. Carbonyl cyanide 3-chlorophyenyl hydrazine (CCCP) is a known MMP disruptor. It was used as the positive control for the loss of MMP (Figure 9A, B). Treatment with PAMAM-O/Flag-apoptin in HepG2 cells showed a lower MMP than that by PAMAM-O/Flag treatment. In contrast, PAMAM-O/Flag-apoptosis did not show obvious changes in MMP in dermal fibroblasts. These results suggest that PAMAM-O/Flag-apoptin treatment of cells causes mitochondrial depolarization, leading to apoptotic death. 

Mitochondria are subcellular organelles surrounded by a double-layered membrane. Mitochondria change in number and morphology via fusion and fission events during apoptotic cell death under various stress conditions. To further confirm the effect of the loss of MMP, we analyzed mitochondrial structural changes using TEM imaging of HepG2 cells and dermal fibroblasts that were transfected with PAMAM-O/Flag or PAMAM/Flag-apoptin and incubated for 48 h. Treatment with PAMAM-O/Flag-apoptin in HepG2 cells showed a significantly altered mitochondrial structure compared to PAMAM-O/Flag treatment (Figure 9C,D). Mitochondria infused with PAMAM-O/Flag-apoptin were swollen in HepG2 cells, whereas PAMAM-O/Flag-apoptin did not appear to cause obvious structural changes in dermal fibroblasts’ mitochondria. Impaired MMP can lead to ROS generation, thereby triggering apoptosis. We found that apoptin-induced cell death was associated with a change in depolarization, which activated the mitochondria-mediated apoptotic pathway in tumor cells.

## 4. Conclusions

Gene therapy is a critical strategy for the effective targeting and specific expression of a therapeutic gene in a tumor region. In the present study, we investigated a gene therapy-based approach using apoptin, a known tumor-selective killer gene, using nonviral gene delivery vectors, namely, PAMAM-O dendrimer in HepG2 cells and dermal fibroblasts. We found that the PAMAM-O dendrimer was non-cytotoxic at various ranges in HepG2 cells. The PAMAM-O dendrimer enhanced transfection efficiency better than the PAMAM dendrimer when the concentration of the polymer increased, and the cells were viable regardless of the polymer concentration. In addition, the polyplexed forms of PAMAM and PAMAM-O dendrimers with Flag or Flag/apoptin were localized in the peri-nuclear region via endocytosis. To improve gene delivery efficiency via endocytosis, the PAMAM-O/Flag-apoptin polyplex was released from the endosome into the cytoplasm, and the polyplexes dissociated, permitting the entry of the apoptin gene into the nucleus across the membrane through the nuclear pore complex. After its expression and accumulation in the nucleus, apoptin can induce apoptosis in cancer cells. Mitochondria-mediated apoptosis was observed after treatment with PAMAM-O/Flag-apoptin in HepG2 cells, but cell death did not occur in dermal fibroblasts. Therefore, we project the PAMAM-O dendrimer as a promising candidate for gene delivery because of its very low cytotoxicity, high cellular uptake, and high transfection efficiency in HeG2 cells. The use of PAMAM-O/Flag-apoptin for tumor-targeting gene therapy requires further studies for applications in clinical settings. Nevertheless, the present results provide a critical foundation for preclinical applications in liver tumor therapy. 

## Figures and Tables

**Table 1:** The characteristics of polymer/Flag or Flag-apoptin polyplexes. Polyplexes were incubated with Flag or Flag-apotin at a weight ratio (polymer:DNA) of 8:1. Data represent mean ± standard deviation (*n *= 3) of three independent experiments.

Polyplexes	Size (nm)	Zeta Potential (mV)
PAMAM/Flag	503.8 ± 11.4	31.1 ± 5.3
PAMAM-O/Flag	181.1 ± 4.0	17.1 ± 1.8
PAMAM/Flag-apoptin	219.1 ± 2.95	35.7 ± 2.2
PAMAM-O/Flag-apoptin	202.1 ± 4.82	14.7 ± 5.3

## Supplementary Materials

The following are available online at www.mdpi.com/2073-4360/9/6/197/s1, Figure S1: Overall synthesis scheme of PAMAM-O, Figure S2. ^1^H-nuclear magnetic resonance (NMR) spectra of (A) PAMAM and (B) PAMAM-O dendrimer.
