# Supplementary material for: Apoptin Gene Delivery by the Functionalized Polyamidoamine (PAMAM) Dendrimer Modified with Ornithine Induces Cell Death of HepG2 Cells
Source: Polymers (Basel). 2017 May 29;9(6):197. doi: 10.3390/polym9060197 (PMC6432117; doi:10.3390/polym9060197)
Supplement: Supplementary file 1 [file polymers-09-00197-s001.pdf]

Supporting information

Supplementary Figures and Figure Legends.

Supplementary Figure S1. Overall synthesis scheme of PAMAM-O.

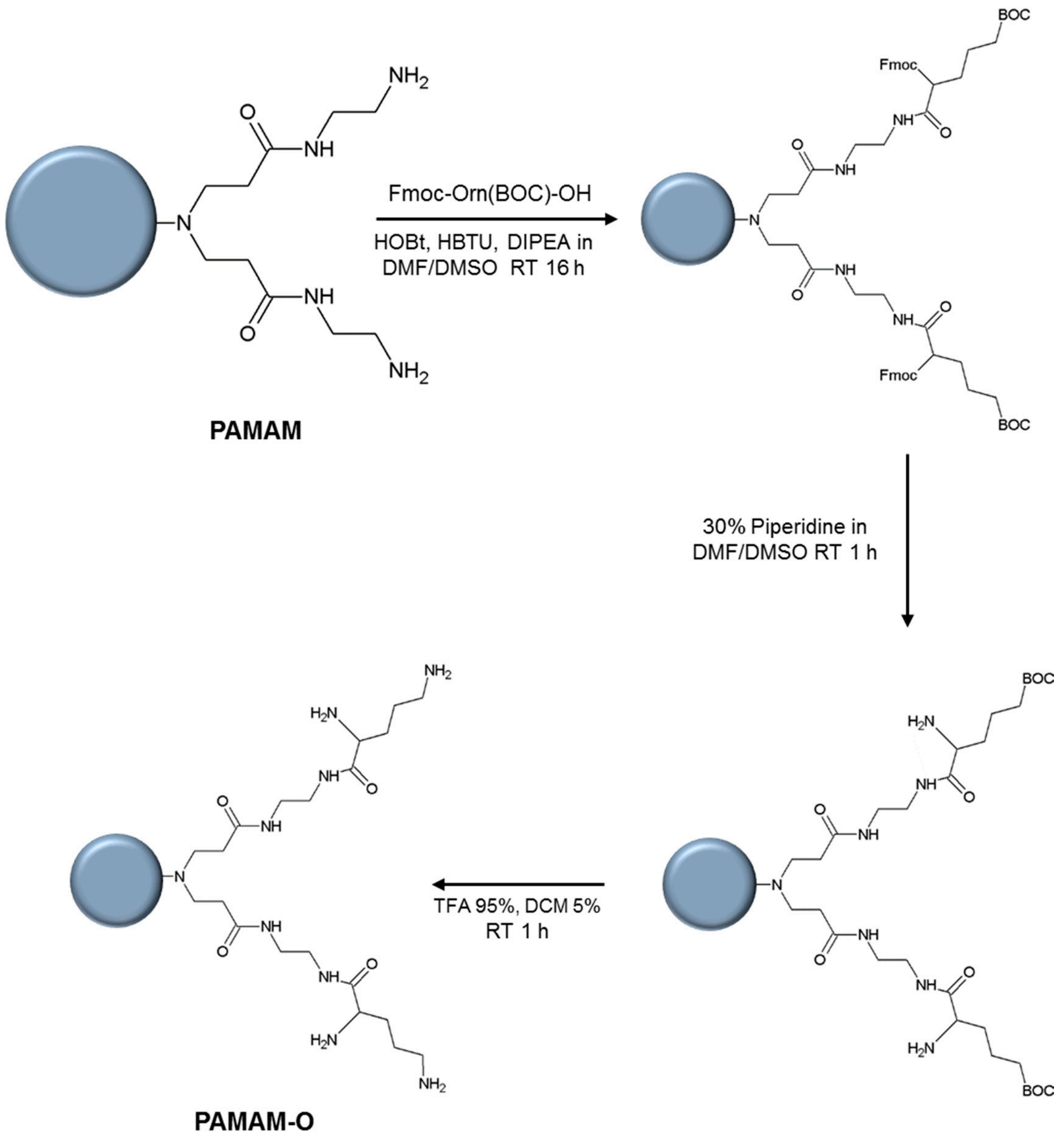

Supplementary Figure S2. ^{1}H-nuclear magnetic resonance (NMR) spectroscopy of the PAMAM-O dendrimer. (A) PAMAM and (B) PAMAM-O.

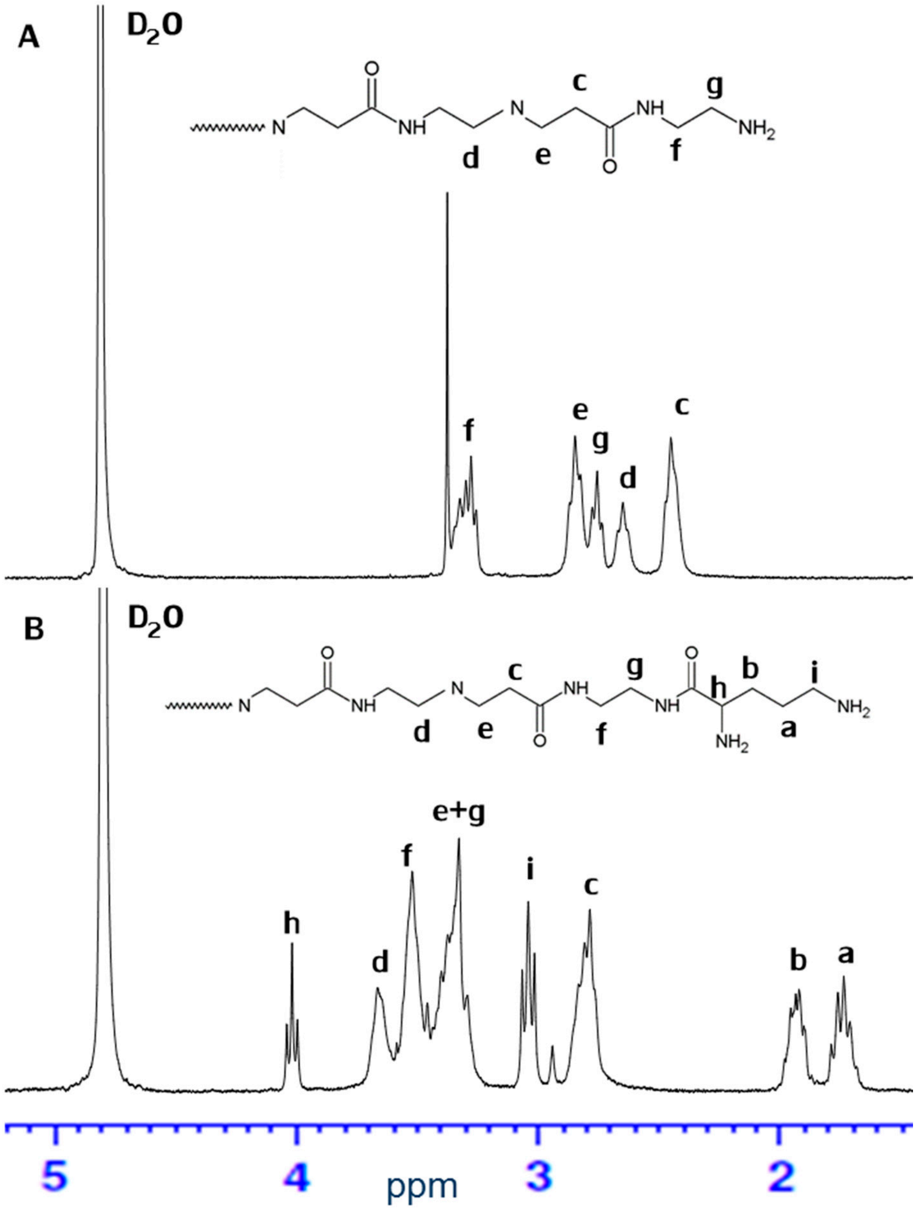

The ^{1}H NMR (300 MHz, D$_2$O) data of the polymers are as follows. (A) **PAMAM:** δ (ppm) 2.44 (-NCH$_2$C***H***$_2$CO- of PAMAM unit), 2.63 (-CONHCH$_2$C***H***$_2$N- of PAMAM unit), 2.83 (-NC***H***$_2$CH$_2$CO- of PAMAM unit), and 3.35 (-NC***H***$_2$CH$_2$CO of PAMAM unit). (B) **PAMAM-O:** δ (ppm) 1.73 (-HCCH$_2$C***H***$_2$CH$_2$NH- of ornithine unit), 1.98 (-HCC***H***$_2$CH$_2$CH$_2$NH- of ornithine

unit), 2.81 (-$NCH_2C\boldsymbol{H}_2CO$- of PAMAM unit), 3.06 (-$HCCH_2CH_2C\boldsymbol{H}_2NH$- of ornithine unit), 3.31 (-$NC\boldsymbol{H}_2CH_2CO$- and -$CONHCH_2C\boldsymbol{H}_2$- of PAMAM unit), 3.54 (-$CONHC\boldsymbol{H}_2CH_2$- of PAMAM unit), 3.68 (-$CONHCH_2C\boldsymbol{H}_2N$- of PAMAM unit), and 4.03 (-$\boldsymbol{H}CCH_2CH_2CH_2NH$- of ornithine unit). The conjugation yield (over 95%) was calculated from the result of ^{1}H NMR.
